# Latest Treatment Option and Technology Advancement in Corneal and Ocular Surface Disease

**DOI:** 10.1155/2014/203868

**Published:** 2014-12-28

**Authors:** Ciro Costagliola, Mark Batterbury, Harminder Singh Dua, Leonardo Mastropasqua

**Affiliations:** ^1^Department of Medicine and Health Sciences, University of Molise, Via F. De Sanctis 1, 86100 Campobasso, Italy; ^2^Ophthalmology Department, St. Paul's Eye Unit, Royal Liverpool University Hospital, Prescot Street, Liverpool L7 8XP, UK; ^3^Academic Ophthalmology, Division of Clinical Neuroscience, B Floor, Eye ENT Centre, Queens Medical Centre, University of Nottingham, Nottingham, UK; ^4^Eye Clinic, University “G. D'Annunzio” Chieti-Pescara, Chieti, Italy

This special issue was focused on the current approaches in the medical and surgical treatment of the most diffuse and important corneal and ocular surface diseases. In addition, the role of current available technologies in the diagnosis and follow-up of patients with ocular surface diseases was also included. We have invited many experts on this topic to highlight the understanding of corneal diseases.

Different topics were exhaustively treated. This allows improving the readers' knowledge on each theme, which is nowadays essential in the correct management of patients.

In detail, M. De Bernardo and coworkers described and discussed the different formulas to overcome the problem of calculating the intraocular lens power in patients that underwent corneal refractive surgery.

L. Mastropasqua and coworkers, focusing on glaucoma surgery, summarized the applications of time and spectral domain anterior segment-OCT in the conjunctival bleb assessment after filtrating surgery. The authors showed the utility of this technology in guiding the clinicians' decisions in the bleb management.

F. Semeraro and coworkers analyzed the potential etiopathogenetic mechanisms involved in the epithelial in growth after Descemet's stripping automated endothelial keratoplasty, reviewing the literature, and discussing the most appropriate therapeutic approaches.

T. Liu and coworkers proposed a relatively safe designed stromal bed thickness to avoid the endothelial damage following lamellar keratoplasty using an Allegretto WaveLight 3 FS200 femtosecond laser.

L. Ambrosone and coworkers evaluated the efficacy of topical verbascoside-based liposomal eye drops in the healing of alkali corneal wound. The authors reported that this approach reduced significantly the first stage of the process of wound healing of the corneal epithelium.

P. Vinciguerra and coworkers compared the biomechanical effect, the riboflavin penetration and distribution in 2 transepithelial corneal collagen cross-linking with iontophoresis (I-CXL), with standard cross-linking (S-CXL) and current transepithelial protocol (TE-CXL), in rabbits. The authors found that I-CXL induced a significant increase in corneal stiffness as well as better riboflavin penetration when compared to controls and TE-CXL, but not to S-CXL.

M. Lanza and coworkers evaluated the correlation between corneal biomechanical and morphological data in healthy eyes, eyes that underwent myopic photorefractive keratectomy (PRK), keratoconus affected eyes, and keratoconus affected eyes that underwent corneal collagen cross-linking (CCC). The authors suggested suggest that corneal curvature would have a greater influence on corneal deformation than corneal thickness.

L. Mastropasqua and coworkers quantified the effect of small incision lenticule extraction (SMILE) on the corneal biomechanics using Scheimpflug noncontact tonometer (CORVIS ST). The authors did not find significant modifications in biomechanical properties after SMILE, suggesting that this procedure could induce only minimal transient alterations of corneal biomechanics.

F. Semeraro and coworkers evaluated the efficacy of 50% autologous serum eye drops in ocular surface diseases not improved by conventional therapy reporting that this treatment effectively stabilized and improved signs and symptoms in patients' affected chemical burns, recurrent corneal erosion, neurotropic keratitis, and keratoconjunctivitis sicca.

A. Hill-Bator and coworkers reported a high cytoprotective ability of trehalose-based eye drops both in viable epithelial corneal cell number after the desiccation and in preservation of cellular functions.

Finally, D. Viswanathan and coworkers evaluated the efficacy of corneal collagen cross-linking for progressive keratoconus in pediatric patients, reporting the treatment as an effective option in stabilizing the condition and reducing the need for corneal grafting.

In summary, the papers published in this special issue confirm the progress in both research and treatment of corneal and ocular surface disease.

The guest editors wish to thank all the authors of this special issue for contributing the high quality papers. We would also like to thank the referees who have critically evaluated the papers.

*Ciro Costagliola* *Mark Batterbury* *Harminder Singh Dua* *Leonardo Mastropasqua*

